# Sauerkraut‐derived LAB strains as potential probiotic candidates for modulating carbohydrate digestion attributing bacterial organic acid profiling to antidiabetic activity

**DOI:** 10.1002/fsn3.4444

**Published:** 2024-10-23

**Authors:** Sujay S. Huligere, Chandana Kumari V. B., Shashank M. Patil, Jayanthi M.K., Ling Shing Wong, Jureerat Kijsomporn, Jameel H. Al‐Tamimi, Ramith Ramu

**Affiliations:** ^1^ Department of Biotechnology and Bioinformatics JSS Academy of Higher Education & Research Mysuru India; ^2^ Department of Pharmacology, JSS Medical College JSS Academy of Higher Education & Research Mysuru India; ^3^ Faculty of Health and Life Sciences INTI International University Nilai Malaysia; ^4^ Nursing School Metharath University Bangtoey Thailand; ^5^ Department of Zoology, College of Science King Saud University Riyadh Saudi Arabia

**Keywords:** antimicrobial, antioxidant, hydroxycitric acid, in silico analysis, lactic acid bacteria, probiotics, RAMULAB48, sauerkraut, α‐glucosidase

## Abstract

Sauerkraut‐derived lactic acid bacterial (LAB) strains have gained attention due to their potential health benefits. This study focuses on evaluating seven Sauerkraut‐derived RAMULAB strains isolated from sauerkraut, aiming to identify promising candidates for modulating α‐glucosidase (AG) and α‐amylase (AM) enzymatic functions. RAMULAB strains with remarkable probiotic potential can contribute to the digestive health and manage conditions like diabetes. Identifying robust candidates from sauerkraut, a fermented food, holds promise for natural and cost‐effective probiotic sources. The RAMULAB strains underwent extensive characterization, including identification through 16S ribosomal RNA (rRNA) sequencing. Their tolerance to harsh conditions, adherence properties, antimicrobial activity, antioxidant potential, and inhibition of AG and AM were assessed. In silico analyses explored their molecular interactions, particularly with hydroxycitric acid, a potential antidiabetic compound. Among the RAMULAB strains, RAMULAB48 emerged as a standout candidate. It displayed exceptional resilience to acidic bile (≥97%), and simulated gastrointestinal conditions (≥95%), highlighting its suitability for probiotic applications. RAMULAB48 exhibited robust adherence properties, including cell‐surface hydrophobicity (80%), autoaggregation (42%), coaggregation with pathogens (≥33%), and adhesion to epithelial cells. Additionally, all seven isolates demonstrated gamma‐hemolysis and resistance to antibiotics (Kanamycin, Methicillin, and Vancomycin), while displaying strong antibacterial properties against foodborne pathogens. These RAMULAB strains also exhibited varying degrees of antioxidant activity, with RAMULAB48 displaying the highest potential (≥41%). In terms of antidiabetic activity, cell‐free supernatant (CS) obtained from RAMULAB48 expressed the highest inhibition levels, notably inhibiting yeast AG by an impressive 59.55% and AM being by a remarkable 67.42%. RAMULAB48 produced organic acids, including hydroxycitric acid (28.024 mg/mL), which showed promising antidiabetic properties through in silico analyses, indicating favorable interactions with the target enzymes. This study identifies *Lacticaseibacillus paracasei* RAMULAB48, a Sauerkraut‐derived RAMULAB strain, as a promising probiotic candidate with exceptional tolerance, adherence properties, antimicrobial activity, antioxidant potential, and antidiabetic effects. The presence of hydroxycitric acid further underscores its potential in managing diabetes.

## Graphical abstract

Molecular modeling studies of organic acid.

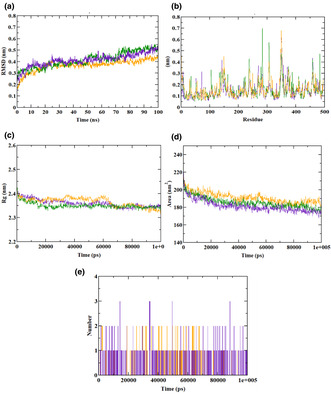

## 1 INTRODUCTION

Diabetes mellitus (DM) is a ubiquitous chronic metabolic disorder brought on by insufficient insulin secretion and/or decreased tissue response to insulin in one or more organs along the complex hormonal pathways. DM has garnered significant attention over the past few decades owing to its rising prevalence and numerous complications (Patil, Manu et al., 2022). The prevalence of DM among people between the age of 20 and 70 years is projected to be 9.6% in India, with 53.1% of the population being undiagnosed, where it is an emerging epidemic, as reported by the World Health Organization (WHO) (Ramu & Patil, 2021). In contrast, 11.3% of the US population is affected by DM, with 23% of adults being undiagnosed, as reported by the Centres for Disease Control and Prevention (CDC), United States of America‐The National Diabetes Statistics Reports (Ramu & Patil, 2021). Approximately 90% of all DM cases are type 2 diabetes (T2D), characterized by insulin resistance, in which the body does not fully respond to insulin, making it the most prevalent type. Numerous factors, such as the family history of diabetes, including rapid urbanization, sedentary lifestyles, meager diets, tobacco use, and rising life expectancy, contribute to the increasing pervasiveness of T2D and other non‐communicable diseases (Patil, Maruthi et al., 2021).

According to a previous research, T2D can be prevented or managed by following a nutritious diet and engaging in regular physical activity (Kumari et al., 2024; Ramu et al., 2022). Over time, people with T2D may eventually require oral medication and healthy lifestyle to keep blood glucose levels under control. Several widely available pharmacological drugs can be used to increase the intensity of DM treatment. These include glucagon‐like peptide‐1 (GLP‐1) agonists, dipeptidyl peptidase (DPP4) inhibitors, and insulin secretagogues (both sulfonylureas and non‐sulfonylureas) (Patil, Kumari, et al., 2024). Biguanides, metformin, and thiazolidinediones are insulin sensitizers. α‐glucosidase (AG) inhibitors delay the breaking down of carbohydrates in the gastrointestinal (GI) tract. Pramlintide, an analog of the peptide amylin, is recommended alongside insulin for both type 1 and type 2 diabetes (Patil & Ramu, 2020a). Even though there are readily available drugs, their consumption over time has been proven to exhibit negative side effects, including renal impairment, cardiovascular illness, appetite loss, fluid retention, and frequent GI tract infections (Patil & Ramu, 2020b; Patil, Sujay et al., 2020). This DM epidemic has continued to increase, despite efforts to find a radical cure. Diverse pathogenic connections are affected by various treatment approaches. However, even when patients achieve ideal glycemic control, there is still a considerable risk of complications. Therefore, this issue requires further research and possibly a fresh perspective on the comprehension and development of new therapeutic techniques.

Gut bacteria have been linked to diabetes and metabolic diseases. T2D patients have a different gut microbiota composition than non‐diabetic individuals, with lower *Firmicutes* and higher *Bacteroidetes* and *Proteobacteria* (Brunkwall & Orho‐Melander, 2017; Egbuna et al., 2021). Subsequently, the GI tract is the host for a variety of microorganisms, and probiotic bacteria are unique among the wide range of microorganisms found in the gut microbiome (Munoz‐Garach et al., 2016). Probiotics are commensal microorganisms found in the GI tract including fermented foods and are Generally Recognized as Safe (GRAS). They have a positive impact on human health by enhancing antagonism against pathogens, promoting nutrient absorption and digestion, enhancing intestinal barrier function, and strengthening the function of the intestinal wall (Qin et al., 2022).

Probiotics improve insulin sensitivity and reduce autoimmune responses by regulating gut microbiota and reducing oxidative stress and inflammatory responses (Feng et al., 2017). They can modify intestinal permeability, influence eating patterns, regulate the gut endocannabinoid system, and control the host's metabolism (Larsen et al., 2010).

Lactic acid bacteria (LABs) are well‐known probiotic microorganisms, playing a crucial role in maintaining health and food production. The discovery of numerous potential health advantages associated with these species has generated a growing interest in them (Li et al., 2017). Fermented foods, dairy products, and animal guts are the most common sources of LAB. LAB strains isolated from all sources have been proven to exhibit various health benefits to the host when ingested as supplements (Dimidi et al., 2019). The consumption of fermented dairy products rich in probiotic LAB is one of the safest and most modern approaches to biotherapy, leading to numerous health benefits, which can be supplemented as food supplements (Tamang et al., 2016). This also acts as an alternative to the direct consumption of dairy products by people who are lactose intolerant (Rivera‐Espinoza & Gallardo‐Navarro, 2010). The administration of cultured probiotic LAB products to lactose‐intolerant individuals has drawn a lot of attention as a result of these observations to minimize the symptoms caused by lactose ingestion (Rivera‐Espinoza & Gallardo‐Navarro, 2010).

Vitamins B and C, dietary fiber, minerals, phytosterols, and phytochemicals are predominantly found in vegetables. Currently, there is an increase in customer demand for ready‐to‐eat foods that taste good, look fresh, have high nutrients, and promote health (Liu et al., 2022). Fresh and barely processed vegetables typically have a short shelf life because of spoilage or contamination by pathogens, which is now a bigger concern for the world's food distribution and economy. To address this issue, fermentation technology has been applied to produce vegetables that are more valuable than raw vegetables by maintaining and improving their safety, nutritional content, sensory quality, and shelf life (Tamang et al., 2009). Chinese immigrants brought cabbage fermentation knowledge to Europe in the 13th century, which produced sauerkraut. The process includes salting cabbage to extract juice, then subject it to spontaneous fermentation. The process is unregulated and has a wide range of microbes in the raw material and processing environment (Zielińska et al., 2015). Pederson (1936) found that northeast sauerkraut fermentation involves heterofermentative LAB (*Leuconostoc mesenteroides* and *Lactobacillus brevis*) that initiate the process and homofermentative LAB (*Lactobacillus plantarum*) that finish it (Pederson, 1936). Starter cultures improve fermented foods by accelerating fermentation, enhancing nutritional value, and altering the characteristics of finished goods. LAB is commonly used for this purpose (Ziarno & Cinchonska, 2021). A study found that using LAB fermentation with *Leu. mesenteroides* and *L. plantarum* improved the quality of traditional Chinese Sichuan Paocai. The fermented Paocai was deemed more suitable for industrial application (Liu et al., 2022).

This study aims to understand the antidiabetic properties of probiotic LAB species isolated from fermented sauerkraut by inhibiting carbohydrate‐hydrolyzing enzymes. The study also included tolerance, safety, exopolysaccharide production, molecular identification, phylogenetic analysis, cell adherence capability, and inhibitory activity of the LAB strains.

## 2 MATERIALS AND METHODS

### 2.1 Bacterial isolation and biochemical characterization

Fresh whole cabbage (approximately 1 kg) was purchased from a local market in Mysuru, Karnataka, India. The outer leaves of the cabbage were trimmed, washed, dried, and diced into small cubes. The diced cubes were placed in a 2‐liter pickle jar along with 6% saline and 2% sugar crystals, until the cubes were submerged under anaerobic conditions. The jar was kept at ambient temperature (25–30°C) for fermentation for a week. The content in the jar was then consecutively diluted and spread‐plated on MRS agar (De Man, Rogosa, and Sharpe agar, HiMedia, Bangalore, India), followed by incubation for 24–48 h at 37°C under anaerobic conditions. Colonies with dissimilar morphological characteristics were selected and tested for Gram‐staining and catalase activity. Only colonies that tested positive for Gram‐staining and negative for catalase were selected and subcultured in MRS broth. These cultures were further used for investigations. Glycerol stock cultures (40% v/v) were prepared for each colony and preserved at −40°C. All the isolates were preliminarily characterized by following the standard guidelines of Bergey's Bacteriology Manual (Vos et al., 2011). The microbial isolates underwent exposure to various environmental conditions, including different temperatures ranging from 4, 10, 37, 45, to 50°C, diverse salt concentrations of 2%, 4%, 8%, and 10%, and a range of pH values, specifically 2, 4, 6, and 8, to assess their tolerance and adaptability under these distinct parameters. The fermentation ability of the isolates was also evaluated using 10 different sugars.

### 2.2 Molecular identification, sequence homology search, and phylogenetic analysis

The identification of Sauerkraut‐derived RAMULAB strains involved molecular techniques specifically targeting the 16s ribosomal RNA (rRNA) region for sequencing. The polymerase chain reaction (PCR) conditions were conducted as per Kumari et al. (2022). To amplify the target region, universal primers 27F and 1492R were employed.

### 2.3 Evaluation of probiotic properties

#### 2.3.1 Tolerance assays

##### Acid–bile conditions

The tolerance of the Sauerkraut‐derived RAMULAB strains to bile acid conditions was assessed with slight modifications based on the methodology described by Huligere, Chandana Kumari, Alqadi et al. (2023). The MRS broth containing 0.3 and 1% oxgall salt concentrations was adjusted to the acidic pH of 2, and broth media RAMULAB culture (100 μL) was inoculated and incubated (at 37°C). Cell viability of the RAMULAB strains was determined at 0, 2, and 4 h after inoculation (Pan et al., 2009).

$$\text{Survival rate}\;\left(\%\right)=\left(\text{Isolate}\;\mathrm{at}\;\text{time}\;\left(t\right)\right)/\left(\text{Isolate}\;\mathrm{at}\;\text{the initial time}\;\left(i\right)\right)\times 100$$

##### Gastrointestinal conditions

The assessment of Sauerkraut‐derived RAMULAB strain tolerance to simulated gastric and intestinal circumstances followed a methodology based on Mantzourani et al. (2019) with minor adjustments. In a controlled in vitro environment, RAMULAB strains at 10^8^ colony‐forming units per milliliter (CFUs/mL) were introduced into gastric and intestinal juice media. Both the gastric and intestinal broths were incubated for 3 and 8 h, respectively, to simulate their respective conditions. The quantification of viable cells was conducted using the colony counting method (Mantzourani et al., 2019).

##### Phenol

The viability of Sauerkraut‐derived RAMULAB strains in the presence of phenolic conditions was evaluated following a method given by Sreepathi et al. (2023). RAMULAB strains, at a concentration of 10^8^ CFU/mL, were exposed to incubation in MRS broth comprising 0.40% phenol for 24 h at 37°C. Following the 24‐h incubation period, the cultured solution was plated on MRS agar and assessed for cell viability through colony counting.

#### 2.3.2 Cell adherence assays

##### Cell‐surface hydrophobicity of RAMULAB

The hydrophobicity of the cell surface exhibited by Sauerkraut‐derived RAMULAB strains was assessed with the methodology described by Martiz et al. (2023).

$$\text{Hydrophobicity}\;\left(\%\right)=\left[1–\left(C/C_{0}\right)\right]\times 100.$$

Where, *C* is the final absorbance at 600 nm of the aqueous phase and *C*_0_ is the initial absorbance at 600 nm.

##### Autoaggregation

A previous methodological approach by Abdulla et al. was used to assess the ability of Sauerkraut‐derived RAMULAB strains for autoaggregation. RAMULAB cultures incubated overnight were resuspended in PBS, and the cell concentration was increased to 1 × 10^8^ CFU/mL. The autoaggregation percentage of RAMULAB was evaluated at intervals of 0, 2, 4, 6, 10, and 24 h (Abdulla et al., 2014).

$$\text{Autoaggregation}\;\left(\%\right)=\left[1–\left(X_{t}/X_{0}\right)\right]\times 100$$

Where *X*_*t*_ and *X*_0_ denote the absorbance at the time “*t*” and “0” (initial), respectively.

##### Coaggregation

According to the previous procedure outlined by Kumari et al. (2023). coaggregation ability between the Sauerkraut‐derived RAMULAB strains and opportunistic pathogens (*B. subtilis MTCC 10403, P. aeruginosa MTCC 98, M. luteus MTCC 1809, E. coliMTCC 4430, and S. typhimurium MTCC 98*) was evaluated at 37°C for after 4 h of incubation (Kumari et al., 2023).

$$\text{Coaggregation}\%=\left[\left(\mathrm{XL}+\mathrm{XP}\right)-X_{\mathrm{mix}}/\left(\mathrm{XL}+\mathrm{XP}\right)\right]\times 100$$

Where *X*_mix_ represents the absorbance measurement obtained from the RAMULAB mixture combined with the pathogen after 4 h, while XL + XP corresponds to the absorbance measurement obtained from the RAMULAB mixture combined with the pathogen at the initial time point, which is 0 h.

##### Adherence to human buccal and chicken crop epithelial cells

The adhesion of Sauerkraut‐derived RAMULAB strains to chicken crop epithelial cells was investigated according to the methodology described by Huligere et al. (2023). RAMULAB strains (100 μL of RAMULAB strains) were mixed with 1 mL of chicken crop epithelial cells (1 × 10^6^ CFU/mL) and incubated at room temperature for 1 h for cell adherence between chicken crop and RAMULAB strains and examined for adherence under a microscope after crystal violet staining. The same procedure was carried out with human buccal epithelial cells to examine the cell adherence potential of the RAMULAB strain.

##### Adherence to HT‐29 cells

The adhesion of Sauerkraut‐derived RAMULAB strains to HT‐29 (human colorectal adenocarcinoma) cells was evaluated, according to the procedure described by Kumari et al. (2023). On a 6‐well tissue culture plate, the HT‐29 cells were seeded at a density of 10^5^ cells/well in a tissue plate and incubated at 37°C for 24 h in 5% carbon dioxide (CO_2_) and 95% air to test the adherence capacity. RAMULAB cultures incubated for 18 h at a cell density of 1 × 10^8^ CFU/mL were suspended in Dulbecco's Modified Eagle's Medium DMEM (Sigma‐Aldrich, India). The wells were thoroughly rinsed three times with phosphate‐buffered saline (PBS) to remove bacterial suspensions and non‐adherent cells. Later on, the MRS agar plate and the final RAMULAB cell viable counts were counted as Log CFU/mL (Kumari et al., 2023).

### 2.4 Safety assessment

In the present study, various assays were conducted to assess the properties of Sauerkraut‐derived RAMULAB strains. The hemolytic activity of these isolates was determined using blood agar plates, following a method described in a previous study (Kumari et al., 2022). Hemolytic activity was gauged by measuring the extent of red blood cell lysis around the colonies, with a clear zone indicating safety. To evaluate antibiotic susceptibility, the disk diffusion method based on CLSI 2018 standards was employed, using a range of antibiotic disks. The results were categorized as resistant (R), susceptible (S), or moderately susceptible (MS) following the Clinical and Laboratory Standards Institute (CLSI) scale (Sreepathi et al., 2023). Additionally, the antibacterial activity of RAMULAB strains against various pathogenic strains was assessed, with slight modifications to the referenced method of Martiz et al. (2023). RAMULAB cultures were inoculated onto Luria‐Bertani (LB) agar plates containing indicator pathogens.

### 2.5 Antioxidant activity

The antioxidant potential of Sauerkraut‐derived RAMULAB strains was evaluated against 2,2‐diphenyl‐1‐picrylhydrazyl (DPPH) and 2,2′‐azino‐bis(3‐ethylbenzothiazoline‐6‐sulfonic acid) (ABTS) radicals at different cell concentrations. The scavenging rate was determined by measuring the absorbance changes in the reaction mixtures, following the method outlined by a previous study (Huligere et al., 2023). The scavenging rate (%) was calculated using the formula: Scavenging rate% = (1 − AS/AB) × 100, where AS represents the absorbance of reactants with the sample and AB is the absorbance of reactants without the sample.

### 2.6 Antidiabetic activities

To evaluate the antidiabetic activities, we adapted methods from previous studies by Huligere et al. (2023) and Kumari et al. (2023). Cell preparation of cell‐free supernatant (CS), cell‐free extract (CE), and intact cells (IC) was followed, as mentioned in Kumari et al. (2022). Both AG and AM assays were conducted according to the previous studies by the authors Kumari et al. (2022). To determine the inhibitory activity of the Sauerkraut‐derived RAMULAB strains, we employed the following equation:

$$\text{Inhibition}\;\left(\%\right)=\left[1–\left(\mathrm{XS}/\mathrm{XC}\right)\right]\times 100.$$

Where XC represents the absorbance of the reactants without the sample and XS represents the absorbance of the reactants combined with the sample.

### 2.7 Organic acid profiling by LC–MS

The CS of the strain with the highest inhibitory activity was used for liquid chromatography–mass spectrometry (LC–MS) analysis. The supernatant was filtered and the filtrate was subjected to solid‐phase extraction once again using a Sep‐Pak C18 cartridge (Kumari et al., 2022). The obtained extract was processed for organic acid profiling by ICAR (Indian Institute of Horticultural Research, Bengaluru) using LC–MS.

### 2.8 In silico assessment of organic acids derived from Sauerkraut‐derived RAMULAB48

#### 2.8.1 PASS pharmacological analysis

The PASS (prediction of activity spectra for biologically active substances) web server was used to examine the pharmacological action. It examines the ability of chemicals to generate a certain pharmacological effect (Patil, Martiz, Ramu, Shirahatti, Prakash, Chandra, et al., 2022). Quantified data were categorized as probability activity (*P*_a_) and probable inactivity (*P*_i_). *P*_a_ values = *P*_i_ are feasible for specific pharmacological properties.

#### 2.8.2 Molecular docking simulation

The *Saccharomyces cerevisiae* isomaltase with a 72% identical and 84% similar sequence at a resolution of 1.8 Å to PDB ID 3AXH (Ganavi et al., 2022) was built using homology modeling. The authors already validated the constructed model in an earlier study (Maradesha, Patil, AI‐Mutairi, et al., 2022). The X‐ray crystal structure of AM (PDB ID:1DHK) was retrieved from Research Collaboratory for Structural Bioinformatics Protein Data Bank (RCSB PDB). The protein preparation (Maradesha, Patil, AI‐Mutairi, et al., 2022; Patil, Ramu, Shirahatti, Shivamallu & Amachawadi, 2021), ligand preparation (Patil, Martiz, Ramu, Shirahatti, Prakash, Kumar et al., 2022), and molecular docking were performed according to the previous studies by the authors (Martiz, Patil, Abdulaziz, et al., 2022).

#### 2.8.3 Molecular dynamics simulation and binding free energy calculations

A 100 ns molecular dynamics simulation using GROMACS‐2018.1 was conducted to evaluate the stability of the protein–ligand complex (Patil, Ramu, et al., 2021). The SwissParam server provides the ligand topology. The system was equilibrated in both NPT (constant number of particles (*N*), constant system pressure (*P*), and constant temperature (*T*)) and NVT (constant number of particles (*N*), constant system volume (*V*), and constant temperature (*T*)) stages for 1000 ps each with 310K temperature and 1 bar pressure (Martiz et al., 2023). Binding free energy calculations were carried out using the Molecular Mechanics Poisson–Boltzmann Surface Area (MM/PBSA) approach g_mmpbsa GROMACS plugin from the trajectories obtained at the end of the simulation. These calculations were performed at the time step of 50 ns, based on the previous study by the authors (Sajal et al., 2022).

#### 2.8.4 Statistical analysis

Experiments were conducted thrice and data were analyzed using analysis of variance (ANOVA), followed by Duncan's multiple range test (DMRT) using SPSS Software (version 21.0, Chicago, USA), identifying significant differences at *p* ≤ .05. Error bars on graphs represent the standard deviation (SD).

## 3 RESULTS AND DISCUSSION

### 3.1 Morphological, biochemical, and genotypic identification

**FIGURE 1:**
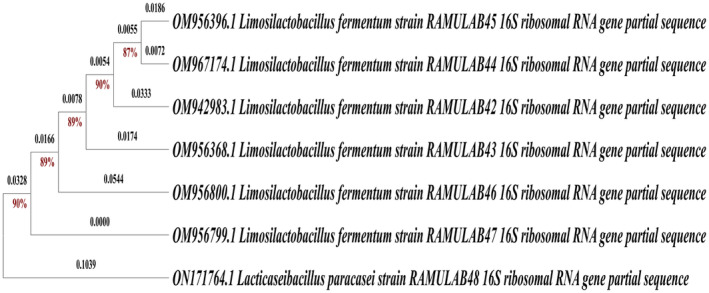
Using maximum‐likelihood bootstrap analysis of 16S rRNA sequences, the phylogenetic tree for RAMULAB strains (RAMULAB42–RAMULAB48) derived from Sauerkraut was constructed.

The selection of potential probiotic bacteria begins with the preliminary characterization of the isolates based on phenotypic, biochemical, and genotypic factors. Seven of the 30 strains isolated from sauerkraut were identified as RAMULAB. All the seven isolates were found to be Gram‐positive and catalase‐negative bacilli by morphology. The seven isolates exhibited positive results for the methyl red test and negative outcomes for the Voges–Proskauer, Indole, and Citrate assays. Additionally, no hydrolysis was evident in the starch hydrolysis assay, and there was an absence of gelatin liquefaction. These isolates demonstrated optimal activity at a temperature of 37°C and a pH of 7, displaying a capacity to tolerate salt concentrations of up to 8%. Furthermore, the fermentation assay revealed the ability to ferment various sugars, indicating a preference for heterofermentation. The biochemical assay results align with those of previous studies, confirming the similar probiotic properties isolated from fermented papaya and fermented pineapple (Martiz et al., 2023; Sreepathi et al., 2023). Genotypic systems are being developed into useful tools that can be applied to a wide range of microbiomes in the modern era (Galloway‐Peña & Hanson, 2020). Identification of probiotic microorganisms by genotypic 16S rRNA gene factors may be more reliable than existing bacterial methods. However, it has been demonstrated that this approach has drawbacks and limitations (Wagner et al., 2003). Because identification is based on a particular sequence homology in comparison with a known database created from previously identified organisms using traditional approaches, speciation depends on the closest match to previously described species in the database (Wagner et al., 2003). Identical sequences can match different taxa with higher levels of homology owing to the database's ongoing growth and change. Consequently, it is currently almost impossible to conclusively determine the speciation of RAMULAB, unless there are extremely well‐characterized isolates (Vicente et al., 2007). Consequently, while 16 s rRNA sequencing can successfully distinguish one RAMULAB isolate from another, its actual accuracy is limited. Comparisons based on homology were arbitrary (Wagner et al., 2003). Despite numerous novel experimental molecular identification methods, sequence analysis of 16S rRNA is the main molecular technique currently available for microbial identification and has proven to be accurate and reliable in databases over time. Table 1 lists the identification scheme of RAMULAB strains (RAMULAB 42–48) with GenBank accession numbers. Biochemically characterized RAMULAB strains were amplified. The sequence length varied from 730 to 1348 bp. The homology search of the sequences RAMULAB42–RAMULAB47 showed >95% similarity to *Limosilactobacillus fermentum*. The sample RAMULAB48 had a similarity of >95% to *Lacticaseibacillus paracasei*, thus validating the isolates sequenced. Of the seven isolates, RAMULAB48 was dissimilar from the other isolates, as shown in (Figure 1). The identification and sequencing results indicate that while RAMULAB42–RAMULAB47 strains align closely with *Limosilactobacillus fermentum*, the RAMULAB48 strain is notably different, being more closely related to *Lacticaseibacillus paracasei*, thus highlighting its unique probiotic potential compared to the other isolates.

**TABLE 1:** Identified RAMULAB strains by 16S rRNA gene sequencing.

Strains	Bacteria	GeneBank accession numbers
RAMULAB42	*Limosilactobacillus fermentum*	OM942983
RAMULAB43	*Limosilactobacillus fermentum*	OM956368
RAMULAB44	*Limosilactobacillus fermentum*	OM967174
RAMULAB45	*Limosilactobacillus fermentum*	OM956396
RAMULAB46	*Limosilactobacillus fermentum*	OM956800
RAMULAB47	*Limosilactobacillus fermentum*	OM956799
RAMULAB48	*Lacticaseibacillus paracasei*	ON171764

### 3.2 Tolerance assays

#### 3.2.1 Acid and bile

**FIGURE 2:**
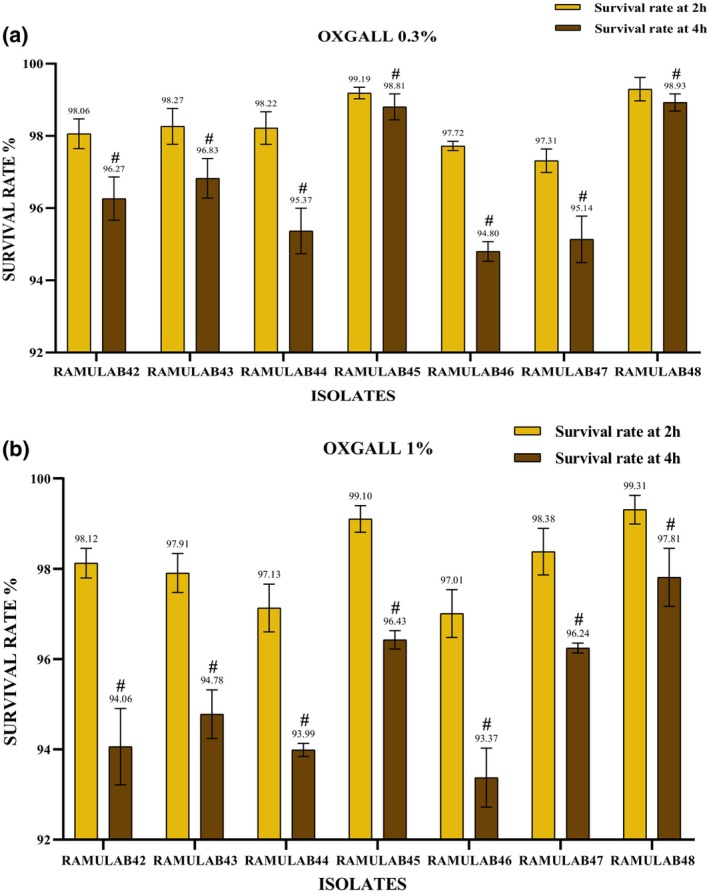
Survival percentage at (a) 0.3% acid and bile salt tolerance, (b) 1% acid and bile salt tolerance. Survival rates at 2‐h intervals are marked with the superscript “#”. A significant difference (*p* ≤ .05) was identified by the Duncan multiple range test (DMRT).

The initial host‐related factors that can impact probiotics are the elevated acidity levels found in both the gizzard and stomach, along with the substantial presence of bile components in the proximal intestine (Pan et al., 2009). Maintaining resilience in acidic environments emerges as a pivotal consideration when selecting probiotic candidates to guarantee their survivability and efficacy (Patel et al., 2006; Mantzourani et al., 2019). Additionally, probiotic bacteria exhibit varying levels of resistance to acidic environments, depending on the species and strain. All seven RAMULAB strains were subjected to 0.3% and 1% oxgall salt concentrations under acidic pH 2 conditions (2 and 4 h at 37°C). (Figure 2a,b) depicts the survival rate of all seven isolates at 0.3 and 1% oxgall concentrations, respectively. All seven isolates demonstrated a survival rate of 94%–99% at 0.3% oxgall, and 93%–97% at 1% oxgall under acidic conditions after 4 h of incubation. Strain RAMULAB48 exhibited the highest survival rate at both concentrations of oxgall. In comparison, bile resistance in our study surpassed the levels reported in prior research by Zielińska et al. for *L. brevis* strain O_2_ (with a survival rate of 97.8% at pH 2.5) (Zielińska et al., 2015). Similarly, when examining gastrointestinal tolerance, our findings exceeded those presented by Romero‐Luna et al. for *L. paracasei* strain CT12, which showed a survival rate of 97.19% ± 3.9% after 30 min in the gastric environment and 42.12% ± 9.6% in the intestinal environment (Romero‐Luna et al., 2020). Remarkably, a recent inquiry into the strains of pickled vegetables has substantiated a strong probability of withstanding the challenges posed by bile salt stress.

#### 3.2.2 Simulated gastrointestinal juice tolerance

**FIGURE 3:**
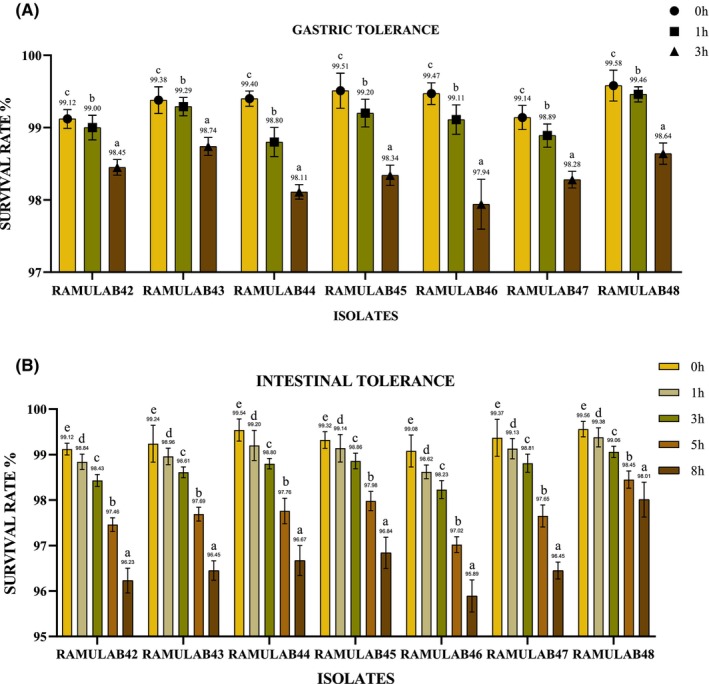
(A) Gastric juice tolerance in percentage. (B) Intestinal juice tolerance in percentage (mean ± SD). A significant difference (*p* ≤ .05) was identified by the DMRT. Denoted superscripts (a–e) survival rates for the time intervals (1, 3, 5, and 8 h).

Probiotic microorganisms must endure the rigorous conditions of the gastrointestinal tract, including the stomach and intestines, to manifest their health‐promoting effects, as highlighted by Reale et al. in their study (Reale et al., 2015). In this study, when considering the entire 3‐h time interval, RAMULAB48 exhibited the highest gastric tolerance, followed closely by RAMULAB45. RAMULAB44 also showed good tolerance but had a slightly lower tolerance at the 3‐h mark. RAMULAB46 consistently demonstrated the lowest gastric tolerance among the isolates across all time intervals (Figure 3A). For intestinal tolerance, overall, when considering the entire 8‐h time interval, RAMULAB48 consistently exhibited the highest intestinal tolerance, followed by RAMULAB45 and RAMULAB44. RAMULAB46 consistently demonstrated the lowest intestinal tolerance among the isolates across all time intervals. Therefore, RAMULAB48 can be considered the RAMULAB isolate with the best overall gastric and intestinal tolerance (Figure 3B). The variations in intestinal tolerance among these RAMULAB strains have significant implications for their potential applications. RAMULAB48, with its consistently high tolerance across all time intervals, emerges as the top candidate for probiotic formulations or therapeutic interventions aimed at gastrointestinal health. Its resilience suggests that it may maintain viability and functionality in the intestinal tract, where probiotics exert their beneficial effects. In the study conducted by Vidhyasagar and Jeevaratnam (2013), it was observed that following a 4‐h incubation period, the population of the candidate probiotic strain *Pediococcus pentosaceus* VJ13 exhibited fluctuations, and its viability experienced a reduction of 20% when subjected to the simulated conditions of gastric and intestinal juices (Vidhyasagar & Jeevaratnam, 2013).

#### 3.2.3 Phenol tolerance

Phenol tolerance in lactic acid bacteria is vital as it ensures their viability and effectiveness in harsh environmental conditions, potentially enhancing their applicability in various industrial and health‐related processes (Gaur & Gänzle, 2023). It is essential to consider that these traits may contribute to the overall functionality and effectiveness of these isolates in various applications, and further research is warranted to explore their specific roles in health‐related contexts (Gebru & Sbhatu, 2020). Regarding the phenol tolerance in our study at 24 h (Table 2), RAMULAB48 displayed the highest tolerance (8.09 ± 0.84 Log CFU/mL), followed by RAMULAB42 exhibited the lowest phenol tolerance (7.44 ± 0.36 Log CFU/mL) among the isolates. In previous studies, *Levilactobacillus* brevis isolated from fermented fruits also showed good tolerance to phenol (Martiz et al., 2023; Sreepathi et al., 2023). Moreover, *Lactobacillus* sp. Strain G3_4_1TO2, which was isolated from bovine colostrum, exhibited a similar phenol tolerance pattern as RAMULAB strains isolated from fermented raw milk, and the rat fecal microbiota (Patterson & Burkholder, 2003; Jena et al., 2013; Padmavathi et al., 2018).

**TABLE 2:** Hydrophobicity, phenol tolerance, and hemolysis of RAMULAB strains from sauerkraut.

Strains	Cell‐surface hydrophobicity (%)^1^	Phenol tolerance as Log CFU/mL^1^	
		0 h	24 h
RAMULAB42	74.80 ± 0.68^b^	7.26 ± 0.12^c^	7.44 ± 0.36
RAMULAB43	70.62 ± 0.12^a^	7.32 ± 0.40^d^	7.78 ± 0.74
RAMULAB44	76.48 ± 0.90^c^	7.24 ± 0.62^b^	7.46 ± 0.41
RAMULAB45	74.97 ± 2.45^b^	7.25 ± 0.11^b^	7.58 ± 0.52
RAMULAB46	68.87 ± 1.90^a^	7.08 ± 0.09^a^	7.46 ± 0.71
RAMULAB47	71.64 ± 0.60^a^	7.28 ± 0.92^b^	7.74 ± 0.57
RAMULAB48	80.42 ± 2.41^d^	7.57 ± 1.02^e^	8.09 ± 0.84

^1^ These values are expressed as means with standard deviations (±) and are accompanied by alphabetical labels (a, b, c, and d) that represent statistically significant differences among the isolates.

### 3.3 Adherence assays

#### 3.3.1 Cell‐surface hydrophobicity

RAMULAB cell‐surface hydrophobicity relies on the hydrophobic constituents found in the RAMULAB's outer membrane, and it plays a crucial role in governing cell adhesion to epithelial cells and the colonization capacity of these isolates (Martiz et al., 2023). In our study, RAMULAB48 exhibits the highest cell‐surface hydrophobicity, suggesting strong hydrophobic properties (Table 2). RAMULAB43, RAMULAB46, and RAMULAB47 have lower hydrophobicity levels. RAMULAB42 and RAMULAB45 show moderate hydrophobicity, with some variability in RAMULAB45. RAMULAB44 also exhibits relatively high cell‐surface hydrophobicity. These findings indicate differences in the surface properties of these isolates, which may have implications for their interactions with host epithelial cells and their potential applications in various contexts, such as probiotics or biotechnological processes. Numerous research findings have consistently indicated that the presence of glycoproteinaceous materials on the cell wall's surface contributes to increased hydrophobicity (Huligere et al., 2023; Kumari et al., 2023; Sreepathi et al., 2023). This hydrophobicity is evident in vitro by the affinity of probiotic strains for chloroform. The current study aligns with these findings, and the results are consistent with those of earlier research (Huligere et al., 2023; Kumari et al., 2023; Martiz et al., 2023).

#### 3.3.2 Autoaggregation

**FIGURE 4:**
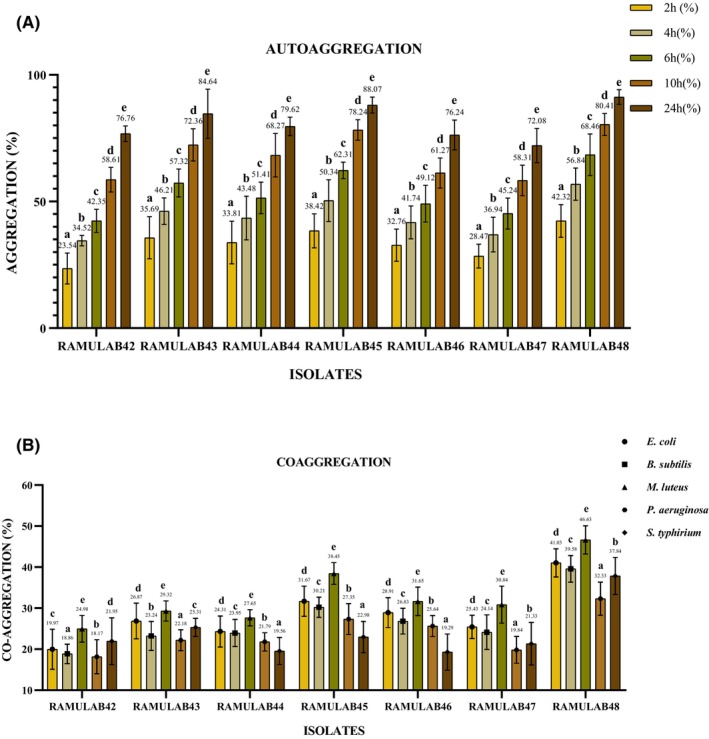
(A) Autoaggregation and (B) coaggregation percentages (mean ± SD) calculated by DMRTs (significantly different [*p* ≤ .05]). Superscripts (a–e) express means of aggregation.

In autoaggregation, where cells of the same strain aggregate, the interpretation remains consistent with a focus on the self‐aggregation behavior of the RAMULAB strains at different time intervals (Figure 4). Autoaggregation can be a crucial aspect of bacterial behavior, affecting biofilm formation, adhesion, and other microbial processes (Tuo et al., 2013). In the current study, isolates at 2 h, self‐aggregation begins, ranging from 23.54% to 42.32%, with RAMULAB48 showing the highest self‐aggregation. As time progresses (4, 6, 10, and 24 h), self‐aggregation increases, with RAMULAB48 consistently displaying the highest levels of self‐aggregation, reaching up to 91.24% at 24 h. This demonstrates that RAMULAB strains have a strong ability to self‐aggregate, with RAMULAB48 leading to this behavior throughout the study. Tuo et al. in their study have shown the aggregation capability of 22 *Lactobacillus* strains, comparatively our strain expressing higher aggregation level with time interval (Tuo et al., 2013). The aggregation can be considered as strain dependent when observed in previous studies (Huligere et al., 2023; Patil, Shirahatti, et al., 2022).

#### 3.3.3 Coaggregation

Coaggregation between probiotic isolates (RAMULAB) and pathogens can occur within the host's gastrointestinal tract. These interactions may influence the ability of probiotics to inhibit the colonization and growth of harmful pathogens. Probiotics that can coaggregate with pathogens may have a higher potential for conferring health benefits to the host by reducing the presence of harmful bacteria in the gut (Hojjati et al., 2020; Reuben, Roy, Sarkar, Alam & Jahid, 2019). The coaggregation percentages with *Escherichia coli* vary among the RAMULAB strains. RAMULAB48 demonstrates the highest coaggregation with *E. coli* (41.03%), while RAMULAB42 has the lowest coaggregation (19.97%). RAMULAB48 also exhibits the highest coaggregation with *B. subtilis* (39.58%), and RAMULAB42 shows the lowest coaggregation (18.86%). RAMULAB48 once again displays the highest coaggregation with *Micrococcus luteus* (46.63%), with RAMULAB42 having the lowest coaggregation (24.98%). RAMULAB48 has the highest coaggregation with *P. aeruginosa* (32.33%), while RAMULAB42 exhibits the lowest coaggregation (18.17%). RAMULAB48 shows the highest coaggregation with *S. typhimurium* (37.84%), while RAMULAB42 has the lowest coaggregation (21.95%). RAMULAB48 consistently exhibits the highest coaggregation with various pathogens. This suggests that RAMULAB48 may have a higher potential as a probiotic strain to compete with and potentially inhibit the growth of these pathogens in the gut. These results can inform the selection of probiotic strains for specific health applications. RAMULAB48, with its strong coaggregation ability, may be considered for probiotic formulations aimed at pathogen control in the gut. The isolate employed in this study aligns with the aforementioned statement, and the observed results are consistent with findings from prior research (Huligere, Desai, Wong, Firdose & Ramu, 2023; Kumari et al., 2022). Furthermore, the study reveals varying degrees of coaggregation, a process that contributes to the maintenance of a harmonious ecosystem within the intestinal environment.

#### 3.3.4 Adhesion ability of chicken epithelial cells, buccal epithelial cells, and HT‐29 cell lines

The isolated chicken epithelial cells were investigated, the adhesion capacity was determined to be 50–100 bacterial cells per epithelial cell, with the least being 20–48 cells. RAMULAB48 had the best adhesion, whereas RAMULAB48 had the least. For buccal epithelial cells, all strains had a similar ability to the chicken epithelial cells.

In Table 3, the values represent the extent to which each RAMULAB isolate adheres to HT‐29 cells, with variations in adhesion percentages observed among the isolates. RAMULAB48 displays the highest adhesion, followed by RAMULAB44, while RAMULAB43 exhibits the lowest adhesion percentage among the isolates tested. The differential adhesion capabilities of RAMULAB isolates, particularly the superior adhesion of RAMULAB48 to HT‐29 cells, highlight its potential application as a probiotic or in gut health interventions, similar to findings by Kumari et al. (2023).

**TABLE 3:** Adhesion capability of RAMULAB strains from sauerkraut to HT‐29 cells.

Isolates	Percentage of adhesion (%)
RAMULAB42	94.17 ± 1.57
RAMULAB43	93.10 ± 0.50
RAMULAB44	97.68 ± 1.20
RAMULAB45	94.53 ± 1.90
RAMULAB46	96.69 ± 2.30
RAMULAB47	95.53 ± 1.53
RAMULAB48	98.21 ± 1.99

*Note*: The values are expressed as mean ± SD.

### 3.4 Safety assessments

It has been postulated that a pivotal factor in pathogen virulence lies in the production of enzymes vital for mucin degradation (Kumari et al., 2023). Consequently, this trait is discouraged in probiotic strains as it can alter the intestinal mucosal lining, rendering it more susceptible to infections and toxins (Huligere et al., 2023). The absence of hemolytic activity is a crucial safety factor for probiotics. Gamma‐hemolysis, signified by the absence of a discernible zone around the colonies, validates the organism's safety. All seven isolates displayed gamma‐hemolysis, characterized by a clear zone, indicative of the absence of hemolysis. Our results coincide with the observations of Oh and Jung, who identified six *Lactobacillus* spp. isolated from fermented millet‐based alcoholic beverages, all of which demonstrated gamma‐hemolysis (Oh & Jung, 2015). The isolates were subjected to testing involving 13 distinct antibiotics to evaluate their antibiotic resistance profiles. These profiles were subsequently compared against the criteria outlined in the reference standard chart (Sreepathi et al., 2023). Sensitivity to antibiotics is a pivotal criterion for a microbe to be considered as a probiotic, making this analysis particularly significant; specifically, seven isolates demonstrated sensitivity to ampicillin (AM), streptomycin (S), rifampicin (R), azithromycin (AZ), erythromycin (E), clindamycin (CLD), tetracycline (T), chloramphenicol (CHL), and gentamicin (GENT), while exhibiting resistance to cefixime (CEF), methicillin (M), kanamycin (KAN), and vancomycin (V) (Table 4). The isolates underwent testing against a variety of foodborne pathogens to assess their antimicrobial capabilities. It is worth noting that in our study, all the isolates displayed antibacterial properties against every tested strain. The diameter of the inhibition zones varied between 6 and 25 mm (Table 5). Across the board, the isolates demonstrated strong antibacterial effectiveness against foodborne pathogens like *M. luteus* and *P. aeruginosa*. Significantly, RAMULAB48 displayed notable antimicrobial efficacy against all pathogens, except *Bacillus cereus, Salmonella, Klebsiella pneumoniae, and Klebsiella aerogenes*, where its inhibitory impact was moderately effective. On the other hand, RAMULAB42 displayed minimal activity against all tested microorganisms, except for *S. aureus*, *P. aeruginosa*, and *M. luteus*, where it exhibited a mild inhibitory effect. This is in line with the *Lactobacillus* spp. isolated from fermented beetroot, dairy samples (milk and curd), cereal batter, and fermented sugarcane juice (Ayyash et al., 2020; Ganavi et al., 2022; Kumari et al., 2022; Huligere, Chandana Kumari, Desai et al., 2023; Kumari et al., 2024).

**TABLE 4:** Antibiotic susceptibility capability of RAMULAB strains from sauerkraut.

Strains	CHL	GENT	CLD	AM	KAN	T	V	E	S	R	M	AZ	CEF
RAMULAB42	−	−	−	−	+	−	+	−	−	−	+	−	−
RAMULAB43	−	−	−	−	+	−	+	−	−	−	+	−	−
RAMULAB44	−	−	−	−	+	−	+	−	−	−	+	−	−
RAMULAB45	−	−	−	−	+	−	+	−	−	−	+	−	−
RAMULAB46	−	−	−	−	+	−	+	−	−	−	+	−	−
RAMULAB47	−	−	−	−	+	−	+	−	−	−	+	−	−
RAMULAB48	−	−	−	−	+	−	+	−	−	−	+	−	−

*Note*: “−” indicates sensitivity and “+” indicates resistance.

**TABLE 5:** The antibacterial capability of RAMULAB strains from sauerkraut.

Strains	*B. cereus*	*S. aureus*	*Salmonella*	*E. coli*	*P. aeruginosa*	*K. pneumo niae*	*M. luteus*	*B. subtilis*	*P. fluores cens*	*K. aerogenes*
RAMULAB42	+	−	+	++	+	+	+++	+	++	+
RAMULAB43	++	++	−	+	++	−	++	++	++	++
RAMULAB44	+	+	−	+	++	++	+++	+	++	+
RAMULAB45	+	++	+	++	++	+	+++	+	++	+
RAMULAB46	−	++	+	++	++	+	+++	++	++	−
RAMULAB47	+	+	+	+	++	−	+++	++	++	++
RAMULAB48	++	++	+	++	+++	++	+++	+	++	++

*Note*: Icons denote areas of inhibition in millimeters: (−) signifies no inhibition; (+) indicates weak inhibition (<7 mm); (++) represents good inhibition (9–15 mm); and (+++) signifies strong inhibition (>15 mm).

### 3.5 Antioxidant activity

**FIGURE 5:**
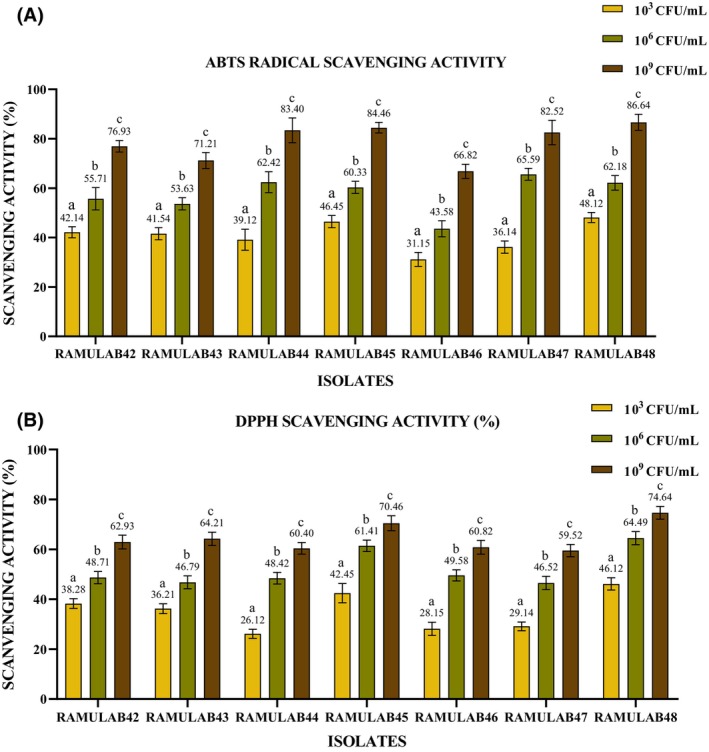
(A) 2,2′‐azino‐bis(3‐ethylbenzothiazoline‐6‐sulfonic acid (ABTS) and (B) 2,2‐diphenyl‐1‐picrylhydrazyl (DPPH) radical scavenging activity percentages (mean ± SD) calculated by DMRTs (significantly different [*p* ≤ .05]). Superscripts (a–c) express means of scavenging activity.

The significance of the antioxidant activity of a drug cannot be overstated, as it plays a crucial role in safeguarding the body against the harm inflicted by free radicals, which trigger oxidative stress (Martiz et al., 2023). RAMULAB strains possess varying degrees of ABTS radical scavenging activity (Figure 5A), which is concentration‐dependent. RAMULAB48 exhibits the highest antioxidant potential, followed by RAMULAB45 and RAMULAB44, while RAMULAB46 displays comparatively lower activity. These findings indicate the potential antioxidant capabilities of the isolates, which may have implications for their use in various applications, including promoting health and preventing oxidative stress‐related diseases. While, results suggest that the RAMULAB strains possess varying degrees of DPPH radical scavenging activity (Figure 5B), which is also concentration‐dependent. RAMULAB48 exhibits the highest antioxidant potential, followed by RAMULAB45 and RAMULAB43, while RAMULAB44 and RAMULAB46 display moderate levels of activity. RAMULAB47 exhibits lower but still notable antioxidant potential. Roughly comparable to the MG860 strain utilized in Kim et al.'s research and surpassing the probiotic strain isolated from kimchi and infant feces as reported by Jang (Ji et al., 2015; Kim et al., 2005).

### 3.6 Antidiabetic activity

**FIGURE 6:**
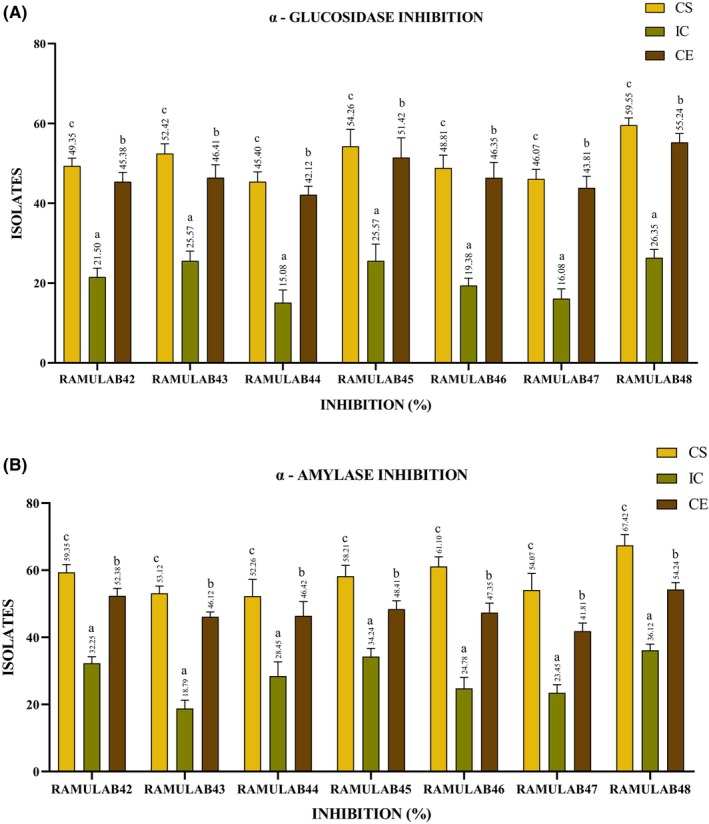
(A) Alpha‐glucosidase (AG) and (B) α‐amylase (AM) inhibitory activity percentages (mean ± SD) calculated by DMRTs (significantly different [*p* ≤ .05]). Superscripts (a–c) express means of inhibitory activity.

Both AG and AM inhibitors are targets for pharmaceutical drugs and natural compounds used to manage conditions like diabetes and obesity (Fujisawa et al., 2005). These inhibitors work by delaying the absorption of carbohydrates, leading to an improved blood sugar control and potentially aiding in weight management (Matsumura et al., 2009). Inhibiting AG can be beneficial in managing blood sugar levels, particularly in individuals with diabetes. Medications or natural compounds that block this enzyme's activity can slow down the digestion and absorption of carbohydrates, leading to more stable post‐meal blood glucose levels (Talamond et al., 2002). AM is an enzyme produced in the salivary glands and pancreas. It plays a crucial role in the initial digestion of dietary starch and glycogen. It breaks down long‐chain carbohydrates into shorter polysaccharides and maltose, a disaccharide (Maradesha,Patil, AI‐Mutairi, et al., 2022). In our investigation, we harnessed the potential of the isolated's derivatives, namely, CS, CE, and IC, to assess their inhibitory activity against AG and AM enzymes. Notably, the isolates exhibited a superior inhibitory capacity when subjected to CS, surpassing the inhibitory effects observed with CE and IC. This inhibitory potential ranged from 15% to 59% with yeast AG and from 18% to 67% for AM (refer to Figure 6). Remarkably, RAMULAB48's CS outperformed the other isolates by achieving the highest inhibition levels, notably inhibiting yeast AG by an impressive 59.55% and AM being by a remarkable 67.42%. This is consistent with the results of earlier studies conducted by Chen et al. (2014). on *L. casei* strain 2W and *Lactobacillus rhamnosus* strain Z7, as well as research conducted by Son et al. (2017). These studies revealed that *Lactobacillus brevis* strain KU15006 displayed potential probiotic characteristics and demonstrated higher AG inhibitory activity in the supernatant (cell‐free) compared to intact cells or extracts. Likewise, similar findings were reported for *L. brevis* strains isolated from fermented sugarcane and fermented fruits (Chen et al., 2014; Martiz et al., 2023; Son et al., 2017; Sreepathi et al., 2022).

### 3.7 Organic acid profiling by LC–MS

*Lactobacillus* strains are renowned for their ability to produce organic acids as part of their metabolic activities. One of the most prominent organic acids generated by *Lactobacillus* is lactic acid, which is widely utilized in various industries, including food and pharmaceuticals, due to its beneficial properties (Patel et al., 2022). This acid is a key end product of sugar fermentation by *Lactobacillus*, contributing to the preservation and flavor enhancement of fermented foods (Nuryana et al., 2019). The RAMULAB48 strain was evaluated for its generation of organic acids, classified as secondary metabolites. The results, outlined in Table 6, revealed the presence of several acids. It aligns with the findings of Kumari et al. (2023), who reported similar organic acid profiles in their *Lactobacillus* isolates, reinforcing the metabolic versatility and probiotic potential of these strains (Kumari et al., 2023).While the primary function of these strains is to metabolize sugar in the medium, producing lactic acid as the main end product, this experiment also detected a substantial quantity of hydroxycitric acid (28.024 mg/mL), surpassing the levels of other secondary metabolites.

**TABLE 6:** List of organic acids extracted from RAMULAB48 strains isolated from Sauerkraut.

CFS bacterial cultures organic acids: molecular formula	RAMULAB48 (mg/mL)
Lactic acid: C₃H₆O₃	8.01
Pyruvic acid: C₃H₄O₃	0.423
Malonic acid: C₃H₄O₄	1.838
Maleic acid: C₄H₄O₄	0.043
Fumaric acid: C₄H₄O₄	0.021
Succinic acid: C₄H₆O₄	4.338
Malic acid: C₄H₆O₅	5.219
Tartaric acid: C₄H₆O₆	0.108
Shikimic acid: C₇H₁₀O₅	0.193
Citric acid: C₆H₈O₇	13.838
Hydroxycitric acid: C₆H₈O₈	28.024

### 3.8 In silico assessment of organic acids derived from RAMULAB

#### 3.8.1 PASS pharmacological potential analysis

According to the results of the PASS evaluation, each of the compounds exhibits strong antidiabetic properties (Table 7). Specifically, hydroxycitric acid and hydroxycitric acid demonstrated high Pa, indicating that they are more likely to be active than other chemicals.

**TABLE 7:** Predicted the PASS result.

Name of the compound	*P*_a_	*P*_i_
Succinic acid	0.440	0.034
Shikimic acid	0.203	0.160
Pyruvic acid	0.228	0.095
Malonic acid	0.270	0.100
Malic acid	0.639	0.009
Maleic acid	0.512	0.021
Lactic acid	0.680	0.007
Hydroxycitric acid	0.708	0.006
Hydroxycitric acid	0.719	0.005
Fumaric acid	0.512	0.021
Citric acid	0.648	0.009
Acarbose	0.693	0.007

#### 3.8.2 Molecular docking studies

**FIGURE 7:**
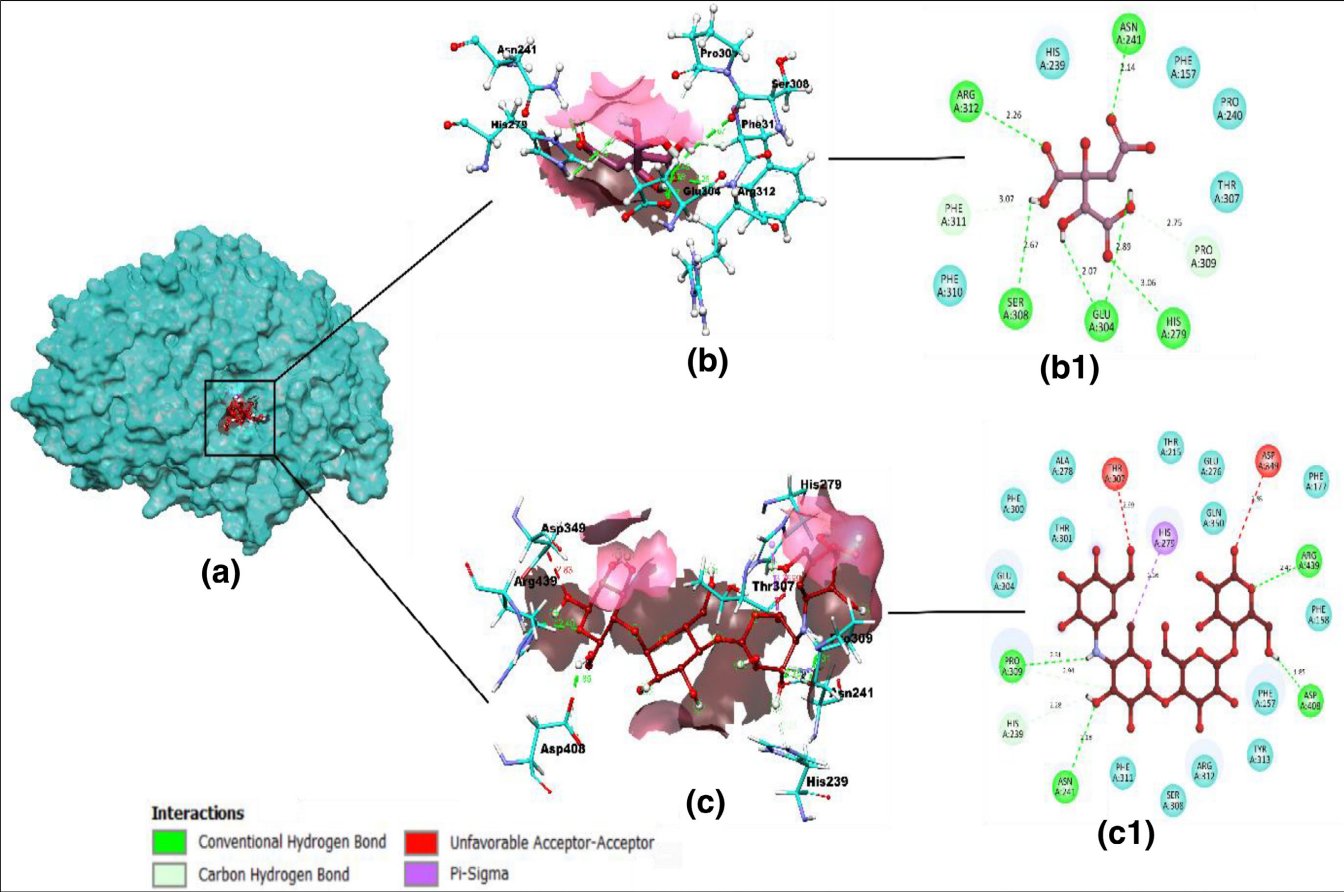
Binding interactions of hydroxycitric acid and acarbose with AG. (a) Surface view depicting the binding site where the ligands bind. (b) Three‐dimensional (3D) view of the binding—spatial arrangement of hydroxycitric acid, (b1) two‐dimensional (2D) view of binding interactions of hydroxycitric acid, (c) 3D view of the binding—spatial arrangement of acarbose, and (c1) 2D view of binding interactions of acarbose.

The results of the virtual screening are given in Table 8. Hydroxycitric acid showed the highest (most negative) binding energy with both AG (−9.7 kcal/mol) and AM (−9.5 kcal/mol). On the other hand, acarbose had a binding affinity of −9.4 kcal/mol with both the target proteins. In a recent study (Maradesha, Patil, Phanindra et al., 2022), the authors identified conserved residues ASN241, ARG312, GLU304, SER308, HIS279, PRO309, and PHE311 in the active site of AG domain A. These residues formed hydrogen bonds with hydroxycitric acid. Conversely, acarbose exhibited seven interactions, including six hydrogen bonds with ASP408, ASN241, HIS239, ARG439, PRO309, and HIS279, a hydrophobic pi–sigma interaction with THR307, and an unfavorable bond with ASP349 (Figure 7). These findings align with prior research by the authors (Patil, Shirahatti, et al., 2022; Martiz, Patil, Thirumalapura Hombegowda, et al., 2022) on AG inhibitor binding interactions.

**TABLE 8:** Virtual screening of *Lacticaseibacillus paracasei* derivatives against AG and AM (PDB ID: 1DHK).

Name of the compound	Binding affinity (kcal/mol)		Total no. of non‐bonding interactions		Total no. of conventional hydrogen bonds	
	AG	AM	AG	AM	AG	AM
Tartaric acid	−9.8	−9.5	8	4	8	4
Succinic acid	−9.1	−8	3	2	3	2
Shikimic acid	−9.3	−9.3	5	3	5	3
Pyruvic acid	−8.5	−6.3	5	2	4	2
Malonic acid	−8.8	−6.8	5	3	5	3
Malic acid	−9.2	−8.4	4	3	4	3
Maleic acid	−9.3	−8.1	5	4	5	4
Lactic acid	−8.5	−6.6	3	4	3	3
Hydroxycitric acid	−9.7	−9.5	7	4	7	4
Fumaric acid	−9.3	−8.2	6	2	6	2
Citric acid	−9.4	−9.4	6	5	6	5
Acarbose	−9.4	−9.4	7	4	6	4

**FIGURE 8:**
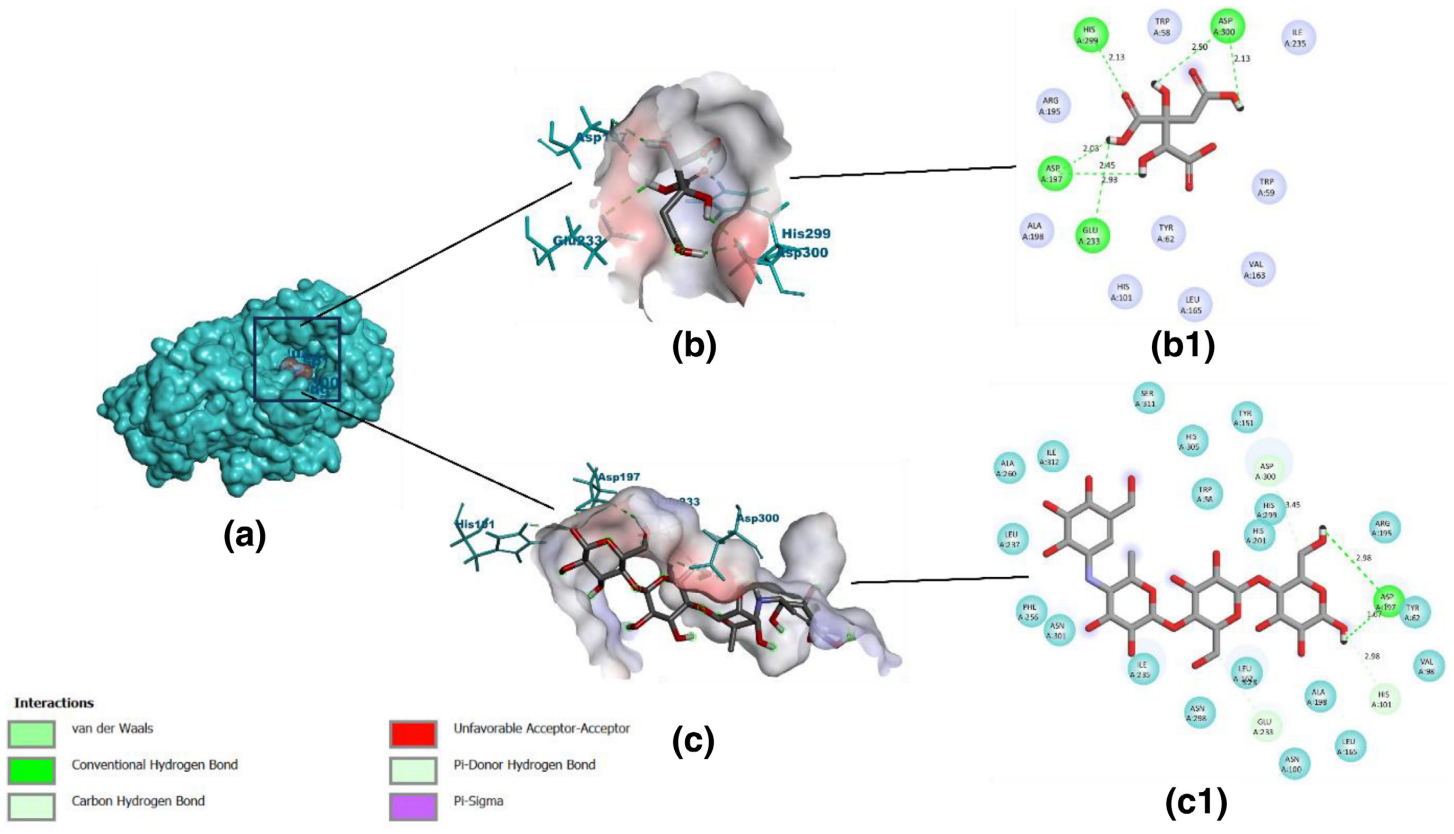
Binding interactions of hydroxycitric acid and acarbose with AM. (a) Surface view depicting the binding site where the ligands bind. (b) 3D view of the binding–spatial arrangement of hydroxycitric acid, (b1) 2D view of binding interactions of hydroxycitric acid, (c) 3D view of the binding –spatial arrangement of acarbose, and (c1) 2D view of binding interactions of acarbose.

In case of AM, both hydroxycitric acid and acarbose occupied the binding site of the co‐crystal ligand (Figure 8). Hydroxycitric acid forms hydrogen bonds with HIS299, ASP300, ASP197, and GLU233. Acarbose, while occupying the same inhibitor‐binding region, establishes three hydrogen bonds with ASP300, GLU233, HIS101, and two bonds with ASP197. However, its larger structure hinders accurate binding, resulting in lower efficiency compared to hydroxycitric acid. The interactions of hydroxycitric acid with AM indicate specific binding to the enzyme's inhibitor site, particularly to catalytically significant residues ASP197 and GLU233 (Ganavi et al., 2022). Therefore, hydroxycitric acid could be an effective inhibitor of AM with a higher binding affinity than acarbose. These results follow the previous studies of the authors (Gurupadaswamy et al., 2022; Maradesha, Patil, Phanindra, et al., 2022).

#### 3.8.3 Molecular dynamics simulation

**FIGURE 9:**
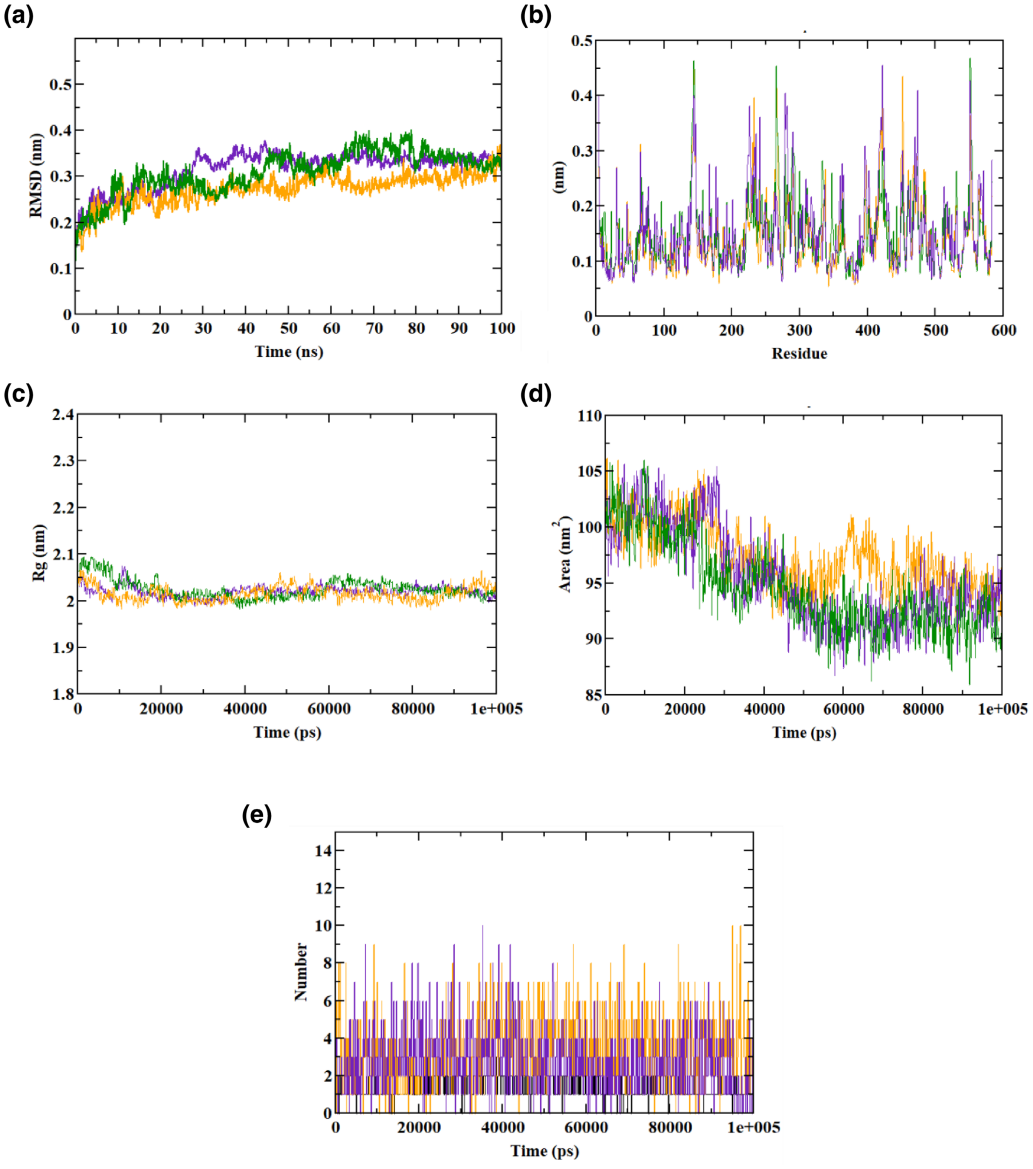
Molecular dynamics (MD) trajectory analysis of AG bound with ligands hydroxycitric acid and acarbose. (a) RMSD, (b) RMSF, (c) Rg, (d) SASA, and (e) ligand H‐bonds. Green: AG apo‐protein, purple: AG–hydroxycitric acid complex, and orange: AG–acarbose complex.

Molecular dynamics (MD) trajectory values for compounds docked with AG are presented in Table 9, with corresponding plots in Figure 9. Root mean square deviation (RMSD) analysis indicates that both the AG–hydroxy citric acid complex and AG apo‐protein reached equilibrium by 50 ns, suggesting the presence of hydroxycitric acid in the inhibitor‐binding site throughout the 100 ns simulation. The AG–hydroxy citric acid complex equilibrated faster than the AG–acarbose complex. Root mean square fluctuation (RMSF) analysis, measuring binding efficiency, showed residues in both complexes fluctuating within the range of 0.1–0.5. The AG–acarbose complex exhibited more variations, indicating instability in the inhibitor‐binding site. Radius of gyration (Rg) plots revealed stability in the structures of AG–hydroxycitric acid and AG–acarbose complexes, with no significant changes during the simulation. These results align with a prior MD study by the authors. (Patil, Shirahatti, et al., 2022). The solvent‐accessible surface area (SASA) plot predicts the occupation of surface area by ligands in the inhibitor‐binding region (Al‐Ghorbani et al., 2023; Pan et al., 2009). Both hydroxycitric acid and acarbose exhibited similar SASA fluctuations in the range of 90–95.5 nm^2^. Additionally, ligand H‐bond analysis indicated that both compounds formed a maximum of 10 atoms during the 100 ns simulation (Shivanna et al., 2022).

**TABLE 9:** Molecular dynamics (MD) trajectory values of hydroxycitric acid and acarbose complexes along with α‐glucosidase.

MD trajectory values	Apo‐protein	Protein–acarbose complex	Protein–hydroxycitric acid complex
Ligand H‐bonds	–	10	10
Rg	2.0–2.05 nm	2.0–2.05 nm	2.0–2.05 nm
RMSD	0.30–0.35 nm	0.35 nm	0.30–0.35 nm
SASA	90–95.5 nm^2^	90–95.5 nm^2^	90–95.5 nm^2^

The RMSD plots for AM depict the AM–hydroxycitric acid complex and the apo‐protein lies at 0.50 nm. However, the AM–acarbose complex was found at 0.45–0.45 nm, slightly below the apo‐protein molecule. Hydroxycitric acid was consistent with low fluctuation during the experiment. The RMSF plot revealed that the AM–hydroxycitric acid complex, AM–acarbose complex, and AM apo‐protein atoms had similar oscillating behavior. Rg plots of all the entities fall within the same 2.35 nm range along with SASA plots of 175–180 nm^2^. The ligand H‐bond showed that hydroxycitric acid had more bonds than acarbose. (Table 10) depicts trajectory values for AM, whereas (Figure 10) depicts the plots of MD trajectories. The results were following the previous studies by the authors (Ganavi et al., 2022; Gurupadaswamy et al., 2022).

**TABLE 10:** Molecular dynamics (MD) trajectory values of hydroxycitric acid and acarbose complexes along with AM.

MD trajectory values	Apo‐protein	Protein–acarbose complex	Protein–hydroxycitric acid complex
Ligand H‐bonds	–	2	3
Rg	2.35 nm	2.35 nm	2.35 nm
RMSD	0.50 nm	0.45–0.45 nm	0.50 nm
SASA	175–180 nm^2^	190 nm^2^	175–180 nm^2^

**FFIGURE 10:**
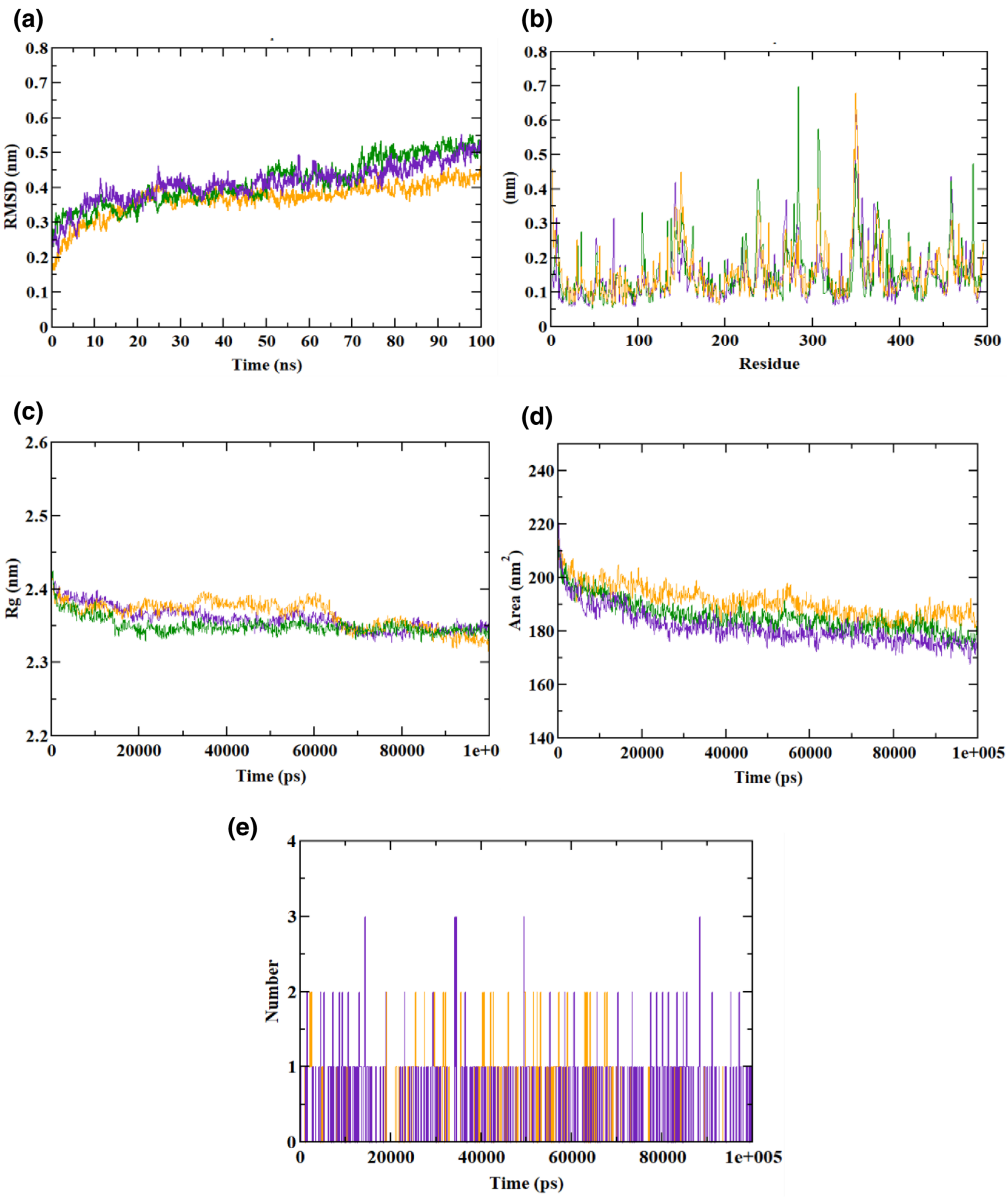
Molecular dynamics (MD) trajectory analysis of AM bound with ligands. (a) RMSD, (b) RMSF, (c) Rg, (d) SASA, and (e) ligand H‐bonds. Green: AM apo‐protein, purple: hydroxycitric acid, and red: acarbose.

#### 3.8.4 Binding free energy calculation

Molecular Mechanics Poisson–Boltzmann Surface Area (MM/PBSA) is a widely used method for computing free binding energies. Analysis of binding free energy highlights the significant influence of van der Waals energy on protein–ligand complexes during MD simulations. Free energy calculations consistently support the energetic feasibility of hydroxycitric acid. In comparison, acarbose‐bound complexes showed lower binding energy than those with hydroxycitric acid, indicating weaker interactions and affinity (Table 11). These findings align with docking and dynamic simulation principles, consistent with prior studies assessing the binding free energy of AG and AM (Al‐Ghorbani et al., 2023; Ganavi et al., 2022).

**TABLE 11:** Binding free energy values of target proteins complexed with hydroxycitric acid and acarbose.

Protein–ligand complexes	Types of binding free energies				
	Electrostatic energy	SASA energy	Polar solvation energy	van der Waal's energy	Binding energy
	(kJ/mol)	(kJ/mol)	(kJ/mol)	(kJ/mol)	(kJ/mol)
AG–hydroxycitric acid	−10.07	−35.78	102.33	−256.09	−201.45
AG–acarbose	−5.98	−12.44	77.11	−125.02	−97.21
AM–hydroxycitric acid	−25.01	−31.07	83.04	−267.08	−195.9
AM–acarbose	−5.2	−10.19	46.5	−128.1	−73.22

## 4 CONCLUSION

In this comprehensive study, seven RAMULAB strains isolated from sauerkraut have been thoroughly evaluated for their probiotic potential and other health‐related attributes. Among them, RAMULAB48 has emerged as an outstanding candidate, exhibiting remarkable resilience to harsh conditions, including acid, bile, and simulated gastrointestinal environments. Its strong adherence properties, such as cell‐surface hydrophobicity, autoaggregation, coaggregation with pathogens, and epithelial cell adhesion, make it a promising choice for probiotic and health‐related applications. The Sauerkraut‐derived RAMULAB strain also displayed significant antimicrobial activity against various foodborne pathogens, with RAMULAB48 standing out for its exceptional performance. Furthermore, these isolates exhibited varying degrees of antioxidant activity, with RAMULAB48 showing the highest potential, suggesting their utility in addressing oxidative stress‐related health issues. RAMULAB48 demonstrated significant inhibitory activity against AG and AM enzymes, highlighting their potential for antidiabetic applications. In silico analyses supported the antidiabetic properties of hydroxycitric acid produced by RAMULAB48, positioning it as a promising compound for diabetes management. The promising attributes of RAMULAB48 warrant further exploration, including clinical trials to validate its probiotic and antidiabetic effects in humans. The implications for food science and nutrition include the potential for incorporating RAMULAB48 into dietary interventions to enhance metabolic health and support diabetes management. Additionally, the strain's metabolic profile offers insights into how specific metabolites might influence glucose regulation and gut health. Also, the production of hydroxycitric acid by this strain opens avenues for research into novel natural antidiabetic agents. Investigating the practical applications of RAMULAB48 in real‐world settings, such as functional foods or therapeutic supplements, will be essential for translating these findings into actionable solutions for diabetes management.

## 5 LIMITATIONS

Our study concentrated on RAMULAB48 and its performance relative to the selected isolates, which may not represent the full spectrum of strains with antidiabetic properties. Future research should broaden the strain evaluation to capture a wider diversity and employ comprehensive metabolomic analyses to uncover additional active metabolites. Additionally, the practical effectiveness of RAMULAB48 in industrial or clinical settings needs further investigation. Expanding the scope and scale of future studies will help validate and extend our findings.

## AUTHOR CONTRIBUTIONS

**Sujay S. Huligere:** Formal analysis (equal); investigation (equal); writing – original draft (equal). **Kumari V. B. Chandana:** Formal analysis (equal); investigation (equal); methodology (equal). **Shashank M. Patil:** Investigation (equal); methodology (equal); visualization (equal); writing – original draft (equal). **M. K. Jayanthi:** Resources (equal); validation (equal); visualization (equal). **Ling Shing Wong:** Supervision (equal); validation (equal); visualization (equal). **Jureerat Kijsomporn:** Resources (equal); software (equal); visualization (equal). **Jameel H. Al‐Tamimi:** Funding acquisition (equal); project administration (equal); supervision (equal); validation (equal). **Ramith Ramu:** Conceptualization (equal); data curation (equal); project administration (equal); writing – original draft (equal).

## CONFLICT OF INTEREST STATEMENT

All authors declare no conflict of interest.

## Data Availability

Not applicable.
